# Aspiration Thrombectomy in a Patient with Suprarenal Inferior Vena Cava Thrombosis

**DOI:** 10.1155/2015/495065

**Published:** 2015-01-27

**Authors:** Hideyuki Kishima, Masashi Fukunaga, Kunihiko Nishian, Ten Saita, Tetsuo Horimatsu, Masataka Sugahara, Takanao Mine, Tohru Masuyama

**Affiliations:** Department of Internal Medicine, Cardiovascular Division, Hyogo College of Medicine, 1-1 Mukogawacho, Nishinomiya 663-8501, Japan

## Abstract

DVT has rarely been observed in the inferior vena cava (IVC). Pulmonary embolism (PE), which can be life-threatening, often occurred in patients with IVC thrombosis. Therefore, an IVC filter is frequently used in those patients for the prevention of PE. A case of successful endovascular treatment of an IVC thrombus in a patient with relative contraindications to implantation of an IVC filter is presented. This case report shows that aspiration of thrombi caught in the removable IVC filter may be an alternative to surgery in high-risk patients with catheter-related suprarenal inferior vena cava thrombosis.

## 1. Introduction

Deep venous thrombosis (DVT) is an important complication in hospitalized patients, but Asian patients have a low incidence of DVT [1]. DVT has rarely been observed in the inferior vena cava (IVC). A recent study reported that DVT occurs in 0.85% of hospitalized patients, and 1.25% of these patients have IVC thrombosis [2]. Moreover, pulmonary embolism (PE), which can be life-threatening, occurred in 12% of patients with IVC thrombosis [2]. Therefore, an IVC filter is frequently used in patients with contraindications to anticoagulation or as an adjunct to anticoagulation for the prevention of PE.

A case of successful endovascular treatment of an IVC thrombus in a patient with relative contraindications to implantation of an IVC filter is presented.

## 2. Case Presentation

**Figure 1:**
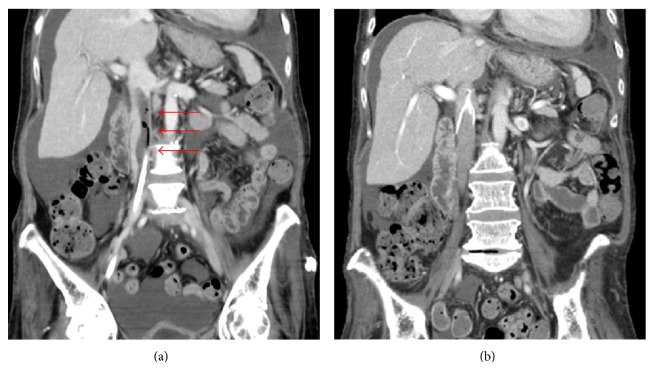
(a) Contrast-enhanced computed tomography (CT) demonstrated nonobstructive thrombus (*arrows*) with air density extending from the CVC via the right femoral vein to the suprarenal IVC, just above the renal vein confluence. (b) Follow-up CT established that there was no IVC thrombus or thrombus in the filter.

A 55-year-old Japanese woman was hospitalized at our institution with recurrent pneumonia. The patient had a history of brain hemorrhage, hypertension, dyslipidemia, thrombotic thrombocytopenic purpura (TTP), right avascular necrosis of the femoral head (ANFH), lumbar canal stenosis, systemic lupus erythematosus (SLE), and recurrent pneumonia due to long-term administration of immunosuppressants and steroids. She received oral tacrolimus hydrate (1.6 mg once daily) and prednisolone (18 mg once daily). She was transported to the hospital with sudden dyspnea. Her physical examination showed a regular pulse rate of 105 beats/min, a blood pressure of 150/100 mmHg, and a normal temperature of 36.6°C. Her pulmonary sounds were diminished in the bilateral lower lung zone. A chest X-ray showed decreased permeability of the lower lung. Laboratory tests revealed an increased D-dimer concentration (5.52 *μ*g/mL) and decreased antithrombin (63%). The patient was negative for antinuclear antibody, lupus anticoagulant, and cardiolipin antibody, and her protein C and S concentrations were normal. Her hospitalization was prolonged due to antibiotic-resistant pneumonia. She needed central venous nutrition, and a central venous catheter (CVC) was inserted via the right femoral vein 34 weeks after admission. At 40 weeks after admission, she developed sudden dyspnea. Contrast-enhanced computed tomography (CE-CT) demonstrated pneumonia, pleural effusion, pulmonary atelectasis, and nonobstructive thrombus with air density extending from the CVC via the right femoral vein to the suprarenal IVC, just above the renal vein confluence (Figure 1(a)). However, no evidence of PE was seen on the CT scan. Echocardiography demonstrated a floating IVC thrombus.

**Figure 2:**
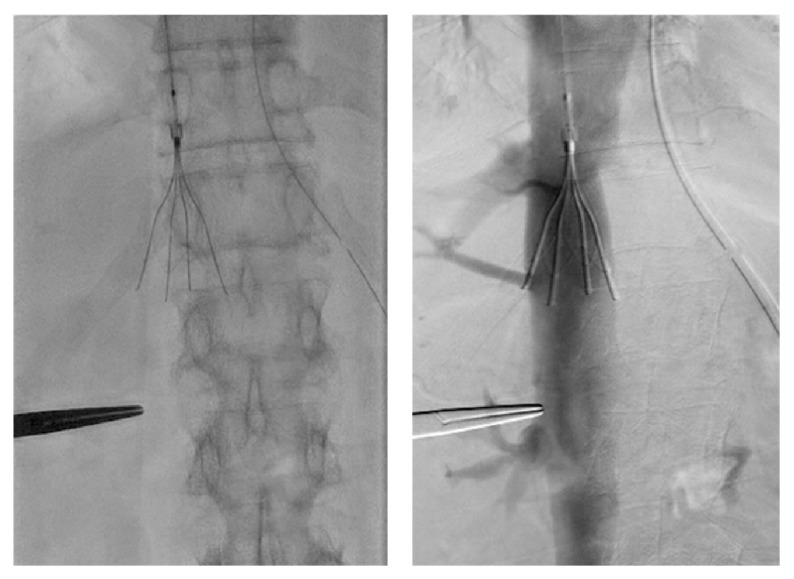
A transient IVC filter was implanted at the site of the suprarenal IVC via the right jugular vein. Insertion of the IVC filter was performed under angiographic and echocardiographic guidance.

**Figure 3:**
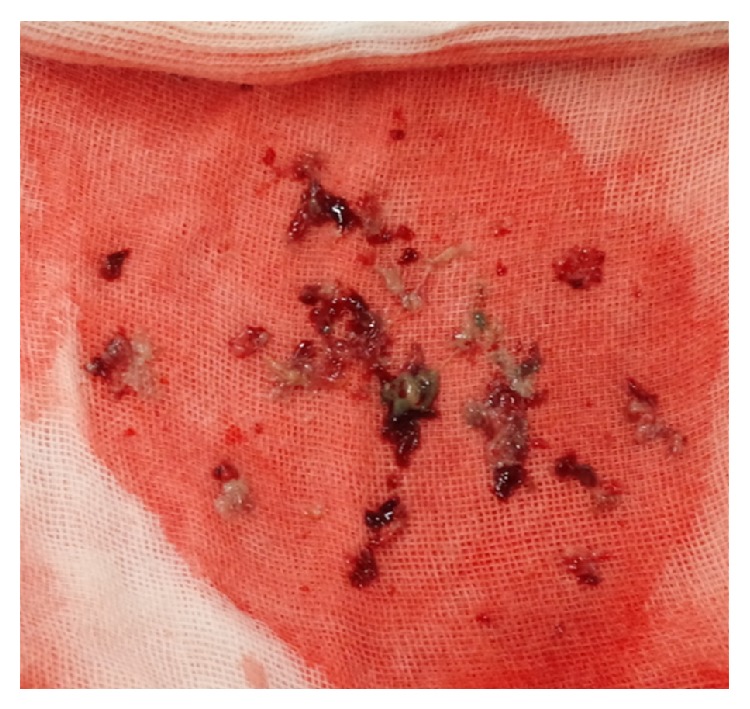
Thrombi caught in the IVC filter were aspirated using a 7-Fr sheath and a 50 mL syringe via the right femoral vein. Many thrombi were removed by the aspiration thrombectomy.

The patient was administered anticoagulant therapy involving unfractionated heparin; however, 6 days later, the IVC thrombus size was not significantly changed. The patient had a small intravascular margin, so she was a relative contraindication for IVC filter implantation. However, the patient was thought to have a high risk of PE and this patient was considered to have a high risk with emergency surgery due to her poor performance status with recurrent pneumonia. Therefore, we decided to implant the IVC filter. A transient IVC filter (Günther Tulip Vena Cava Filter, Cook, Bloomington, IN) was implanted at the site of the suprarenal IVC via the right jugular vein. Insertion of the IVC filter was performed under angiographic and echocardiographic guidance (Figure 2). A CVC and a thrombus with air density can become possible causes of infection; therefore, the CVC was removed; thrombi caught in the filter were aspirated using a 7-Fr sheath and a 50 mL syringe via the right femoral vein. Many thrombi were removed by the aspiration thrombectomy (Figure 3). Since it was clear that a suprarenal IVC thrombus and thrombus in the filter were not present, the IVC filter was repositioned at the site of the infrarenal IVC. Echocardiography of the IVC showed no thrombus at the last examination.

The patient was treated with an oral anticoagulant. Microbiologic study of the thrombus did not reveal infection. No evidence of PE was seen on the follow-up CT and lung ventilation-perfusion scans 2 weeks after the procedure. The follow-up CT (Figure 1(b)), echocardiography, and angiography established that there was no IVC thrombus or thrombus in the filter, and the IVC filter was then removed. It was thought that the patient had a low risk of fatal PE; therefore, she was moved to the general ward, and the treatment of pneumonia was continued.

## 3. Discussion

The occurrence of IVC thrombosis may generally be related to autosomal dominant polycystic kidney disease, pancreatitis, ovarian cysts, renal cell carcinoma, posttraumatic hematoma, chemotherapy, radiotherapy treatment, or liver abscess [3, 4]. A CVC may also promote IVC thrombus formation. Warden et al. studied 139 patients with a CVC. IVC thrombus was detected in 51 patients (36.7%) at autopsy [5]. Chastre et al. reported that IVC thrombi were detected in more than half of patients with a CVC, but few complications were noted after CVC removal [6]. However, the risk of PE has been shown to be higher in patients with free-floating thrombus [7]; therefore, aspiration thrombectomy was performed after implantation of the IVC filter in the present case. Surgical removal of the thrombus could not be performed because the patient had concomitant pneumonia.

According to Japan Circulation Society (JCS) guideline, a small intravascular margin is regarded as a relative contraindication for IVC filter implantation. Previous reports showed that there was an increased risk of decline in renal function [8], filter migration [9], and fracture [10] with implantation of an IVC filter at the suprarenal IVC. This IVC filter was approved for an IVC of 30 mm, and this case's IVC diameter was within 30 mm on the preprocedural CT. However, the diameter of the IVC is related to venous return, blood volume, and the respiratory cycle. Compared with an infrarenal IVC, a suprarenal IVC is larger in diameter but shorter in length. Matchett et al. reported a 3% rate of migration with suprarenal filters [9]. If a patient experiences filter migration, emergency surgery may be needed. The present patient was considered to have a high risk with emergency surgery due to her poor performance status with recurrent pneumonia. Therefore, filter implantation was performed with an IVC filter with echocardiographic guidance. A migration risk-free environment could be ensured with this procedure.

This case report shows that high-risk patients with catheter-related inferior vena cava thrombosis should undergo echocardiography and CT before catheter removal. If there is a free-floating IVC thrombus, temporary removable IVC filter placement with echocardiographic guidance and aspiration thrombectomy may be a valuable alternative treatment.

A case of successful endovascular treatment without causing PE in a patient with catheter-related free-floating IVC thrombosis was described. Aspiration thrombectomy using a removable IVC filter may be an alternative to surgery.
